# Operative Dentistry

**Published:** 1890-10

**Authors:** Fred D. Fisher


### THE DENTAL REGISTER.

Vol. XLIV.] OCTOBER, 1890. [No. 10.

### COMMUNICATIONS.

### Operative Dentistry.

BY FRED D. FISHER, D.D.S.

Defects in *enamel* which are liable to occur during development of the teeth are in the form of chalk-like spots, pits, or grooves, transverse corrugations and fissures in the masticating surfaces of molars and bicuspids where the enamel has failed to unite.

The white chalk-like spots may be found on any part of the tooth or teeth, not necesarily upon all the teeth in the same mouth; they will usually be found softer than the surrounding enamel.

The pits and grooves may appear on any part of the teeth, but are usually found on the labial and lingual surfaces, especially on the anterior superior teeth; they may reach right through the enamel to the dentine or only part way. Sometimes the pits are arranged in regular order in a horizontal line in such a manner that if the septa of enamel between them be removed they would form a groove.

Transverse corrugations of the enamel are looked upon, and most likely are, defects in the structure. They are usually found on the labial surfaces of the six anterior teeth, also, but perhaps more rarely, on the bicuspids and molars. The edges of the enamel sometimes fail to unite on the masticating surfaces of the molars, the bicuspids forming fissures which may reach down to the dentine, or only partly so. All these several conditions are classed under the head of *Atrophy*.

The defects in the dentine are not so numerous as in the enamel. The most common is that imperfectly calcified form of dentine known as the interglobular spaces. These occur between the periphery of the dentine and the wall of the pulp cavity. They usually are arranged somewhat after the shape of the tooth.

The granular layer of Purkinje lies between the dentine and enamel and is considered by most authors to be due to arrested development. It is less calcified than the rest of the dentine and more readily attacked by decay.

The conditions or influences liable to produce defects in enamel and dentine during development and growth are eruptive fevers, measles, small pox, scarlet fever, etc.

During the embryonic stage a severe illness of the mother would be likely to leave its impress upon the teeth of the child. The child may not be sufficiently nourished during the period of tooth development, or having sufficient food some derangement of the assimilative or metabollic process may result in the food not being built up properly into the growing tissues.

Any systemic disease during childhood will be likely to leave its impress upon the teeth. Three of the most common diseases of the tooth pulp are the following:

1. Inflammation. This is by far the most common disease of the tooth pulp. It goes through the stages of inflammation in a similar manner to other tissues of the body with the exception of swelling and pain; being confined in a resistant bony canal it has no room to swell, except when exposed, at the orifice of exposure. This accounts for the pain which is out of all proportion to the size of the tissue inflamed and extent of inflammation.

The color of an inflamed pulp varies with the stage of inflammation from bright red to reddish-brown.

It is not necessary that a pulp be exposed before it can take on inflammation; constant irritation from sensitive dentine, or great thermal changes.

Treatment. Antiphlogistic.

To obtain the best results treatment should be both local and systemic.

SYSTEMIC. Place the system in a good condition; if hyperæmic give saline cathartics to divert the blood from the head.

LOCALLY. Remove the cause; remove all irritants; cleanse out the cavity of decay; make surrounding parts thoroughly antiseptic. The pulp may sometimes be depleted by puncturing.

If the surface is inclined to suppurate, or become necrosed, it may be digested off with pepsin until healthy tissue is reached. Apply a little pepsin paste (pepsin powder and HCl.) seal up the cavity and allow it to remain for a few days.

When the pulp has been brought into a good condition it may be capped and the cavity permanently filled.

The best method of capping is to take a sufficient quantity of powdered oxide of zinc, mix into a paste with some antiseptic, non-irritating liquid, as glycerine, water with a small per centage of carbolic acid; flow this paste gently over the surface of the pulp; remove excess of liquid by some absorbent material; lay over this a layer of oxyphosphate cement, and proceed to fill permanently. Caps of metals such as lead, platinum, etc., have been used.

2. HYPERTROPHY OF THE PULP. Sometimes from long-continued inflammation the pulp becomes hypertrophied and spreads out into the cavity of decay. The hypertrophied portion is usually without nerves, but very vascular; its size will vary with the duration of the inflammation causing it.

TREATMENT. EXCISION.

With a sharp instrument the growth is carefully removed, as little irritation being given to the pulp as possible; the cut surface at the orifice of exposure may be cauterized, and a scar produced by applying nitric acid, nitrate of silver, tannic acid, or the actual cautery. Arsenious acid should not be used. An eschar having been produced the cavity may be filled.

3. NECROSIS OF THE PULP. Death of the pulp may occur from one of several causes, as the result of an inflammation, from sudden blows, or changes of temperature, etc. The anterior teeth are most liable to this affection, being more exposed than those further back in the mouth. Death of the pulp is usually preceded by violent pain. Sometimes, though rarely, the portion of the pulp occupying the crown will be necrosed whilst the remainder is alive.

TREATMENT. REMOVAL.

Dead pulp must be thoroughly removed and the pulp cavity rendered antiseptic. Any portion that may be allowed to remain is liable to putrefy and cause periostitis, etc. The pulp canal must be thoroughly sealed with an aseptic material.

4. SUCCESSIVE STEPS IN THE FORMATION OF AN ACUTE ALVEOLAR ABSCESS.

1. IRRITATION. Irritation presupposes an irritant, which in this case may be inflammation of the pulp, necrosed pulp, exostosis, calculus, etc. The irritation produces a disturbance in the blood flow resulting in

*Determination* of blood to the part; this extra flow of blood causes increased heat and causes dilatation of the vessels which tends to increase the size of the parts, but in the tooth socket there is little room for swelling, so the tooth is often slightly displaced. For a time the flow of blood is more rapid, but after a while the white corpuscles begin to cling to the sides of the vessels, and gradually there is a damming up process called *stasis.* This is rarely complete as the blood in the centre still continues to flow rapidly. The next step is

*Infiltratiou;* the white corpuscles and blood plasma are exuded through the walls of the vessels into the surrounding tissues, causing swelling. The tissues in the infiltrated area now begin to break down and cell proliferation takes place rapidly forming pus; the formation of pus being ushered in by a chill. To make room for the pus in the tooth socket the alveolus is absorbed and the pus thus gradually works to the surface in the direction of least resistance. The pus cavity is surrounded by a layer of coagulated lymph, showing an attempt of nature to reorganization.

PRINCIPLES OF TREATMENT.

Remove the cause.

Get rid of the inflammation by local or general treatment.

Evacuate the pus and thoroughly cleanse the cavity, if there is any serumnal calculus on the roots it must be carefully removed by scraping, by dissolving with sulphuric acid, or by the use of a small engine burr. If there is any necrosed alveolus it must be thoroughly removed.

The layer of coagulable lymph must be also removed by dissecting.

The parts must be *made* and *kept* thoroughly antiseptic.

5. DIFFERENCES BETWEEN CHRONIC AND ACUTE ALVEOLAR ABSCESS. All the degenerative processes are more marked in chronic abscess; there is greater destruction of tissue; more pus; greater deposition of serumnal calculus on the roots; more absorption of the alveolus. In chronic abscess the disease may have spread and involved other teeth. The recuperative power of the parts is lower and treatment must be long-continued.

Chronic abscess has a greater effect on the general system from absorption of pus and general disturbance.

6. THEORY OR PRINCIPLES OF THE TREATMENT OF INFLAMMATION.

1. To get rid of the cause of irritation; it is scarcely, if at all, possible to permanently cure an inflamed tissue whilst the irritant is allowed to remain.

2. *Antiphlogistic.* There is a hyperamic condition of the affected tissue which must be removed. There are several ways to do this. One is through the general system by the use of cathartics and cardiac sedatives. Another is locally by contracting the vessels, using cold, astringents, etc.

By counter irritation, in order to draw the blood to some other part where it will do less harm.

By *depletion*, removal of blood by cupping, scarifying, etc. Rest and quiet to the affected tissue.

7. SUCCESSIVE STEPS IN THE INTRODUCTION OF A GOLD FILLING.

1. Examination of the case. The case must be thoroughly examined; note the character of the tooth, of the carious cavity, and of the decay. Note the occlusion of the opposing teeth, especially if such occlusion would affect the filling.

2. Clean the teeth. A filling should never be introduced into a tooth covered with calculus and dirt. Polish the enamel.

3. Remove the decay; where there is little flow of saliva this may be done before the rubber cloth is applied. If there is a great free flow of saliva the rubber cloth must be first applied. Care should be taken to remove all the decay unless such removal would weaken the walls of the cavity, or expose the pulp. The harder varieties of decay may sometimes be allowed to remain as a covering for the pulp, but must first be made thoroughly antiseptic.

4. Antisepticise the cavity.

5. Dry the cavity thoroughly.

6. If the form of the cavity after the removal of decay is not sufficient to retain the gold, or to allow of its free adaption it should now be given that form, care being taken not to weaken the walls or expose the pulp.

7. Trim the borders of the cavity, giving them as much strength as possible. Make them even and smooth.

8. Examine the prepared cavity to make sure it is in a proper condition for the introduction of the gold.

9. Introduce the first piece of gold (preferably soft) to the bottom of the cavity in such a situation that it will be properly retained; be sure that it is firmly fixed in position. Never allow a piece of gold to remain if it rocks or moves.

10. Proceed with the introduction of gold in small pieces taking care that each piece coheres perfectly, and that the gold is thoroughly adapted to the walls of the cavity. It is better to keep the sides of the gold a little higher than the centre. Be careful to have a good receiving surface.

Extra care is necessary when adapting the gold to the orifice of the cavity, especially at the cervical margin.

Thoroughly consolidate each piece of gold, especially at the surface.

Finish the filling, giving it the natural contour of the tooth, making sure that no thin edges of gold overlap the enamel and that there is no place left where food can lodge.

Examine the filling and see that it is perfect.

### Three Diseases of the Gums.

1. *Tumid Gums.* Gums red, swollen, sensitive, bleed readily, excessive secretion.

2. *Hypertrophied Gums.* Gums firm, inelastic, more or less increased in size, not very sensitive, bleed readily, may partly or totally cover the teeth.

3. *Inflammation of the Gums.* Red, swollen, sensitive, surface dry and shining, very painful.

